# Editorial: Effect of Genotype and Pre- and Post-harvest Factors on Volatile Organic Compounds, Nutritional and Sensorial Quality of Fruits and Vegetables

**DOI:** 10.3389/fnut.2022.945170

**Published:** 2022-06-07

**Authors:** Bernardo Pace, Maria Cefola, Francesco Di Gioia, Rosaria Cozzolino

**Affiliations:** ^1^Institute of Sciences of Food Production, National Research Council of Italy (CNR), Foggia, Italy; ^2^Department of Plant Science, Pennsylvania State University, University Park, PA, United States; ^3^Institute of Food Science, National Research Council (CNR), Avellino, Italy

**Keywords:** pre- and post-harvest treatments, genotype, volatile organic compounds, packaging, fruit and vegetable

Five papers were published covering the themes of the editorial Research Topic: pre-harvest applications, post-harvest treatments, modified atmosphere packaging, non-destructive tools, and identification of volatile organic compounds (VOCs) as markers of freshness, origin, and storage conditions. Hereafter are highlighted the main findings of the studies collected through this Research Topic.

Michailidis et al. described the effects of exogenous melatonin applications, through pre-harvest foliar spray and/or postharvest immersion, on sweet cherry (cv. Ferrovia) fruit. The effects on the ripening parameters of fruit and their relationship with bioactive compounds and gene expression at harvest, after storage at 0°C for 12 days followed by 8 h at 20°C were evaluated. Results illustrate that combining pre- and post-harvest treatments, melatonin played an important role in many biological processes, including the depression of chilling injury symptoms, the delay in ripening and the decay incidence. Moreover, melatonin activated the antioxidant and secondary metabolism. Analyzing the sweet cherry phenolic profile at harvest and after cold storage via UPLC-MS/MS, 28 phenolic compounds, remarkably induced by melatonin at harvest and especially following cold treatment, were identified and quantified. Melatonin application caused phenolic compounds accumulation, including proanthocyanidins and anthocyanins, through the up-regulation of various genes related to phenols biosynthesis at harvest and, particularly, after cold exposition.

On-tree, fruit respiration was depressed by the pre-harvest spray of melatonin, while combined pre- and postharvest treatments delayed fruit softening during storage. These findings also highlight that sweet cherry response to melatonin involved both a cold-dependent activation of the respiration and an up-regulation of the tricarboxylic acid cycle genes.

The second paper was aimed to explore the applicability of an electronic-nose (E-nose) as a rapid method for discriminating samples of sweet cherry cv “Ferrovia” packaged in high-CO_2_ or air for up to 21 days (Cozzolino et al.).

Projection to Latent Structures methods applied to E-nose data showed that fresh fruit and the stored samples can be distinguished according to both the condition and the days of storage. Correlation analysis between E-nose sensors and 45 VOCs, detected by HS-SPME/GC-MS from all the investigated sweet cherry samples, allowed to associate a specific volatile profile to one or more E-nose sensors. The VOCs detected can be considered putative markers of freshness and might be used for a rapid assessment of the product quality. Finally, several quality attributes were investigated during storage. Among them, visual quality and berry deformation resulted affected by storage conditions, showing that high-CO_2_ treatment better preserved sweet cherry quality during the storage.

In the third paper, Antoniou et al. analyzed the volatile profile of carob powder milled of two genotypes cultivated at different altitude (15 and 515 a.s.l.) and harvested at six maturity stage. Fifty-six VOCs including acids, esters, aldehydes, ketones, alcohols, furans and alkanes were identified by HS-SPME/GC-MS analysis. During ripening, the most abundant volatile was isobutyric acid, providing the characteristic cheesy-acidic-buttery aroma of carob, while aldehydes and alcohols decreased, lessening the green grassy notes. At the immature stages, a pleasant aroma, attributed to isobutyrate and methyl isobutyrate esters, was detected, imparting to the unripe green carob powder a potential admixture component for improving the aroma of novel food products. At low altitude the acids, mainly of less pleasant aroma, and all the esters showed the highest value. Isobutyric acid was more abundant at higher altitude, indicating that cultivation at these altitudes can enhance the characteristic carob-like aroma and sensory qualities of carob powder. On the other hand, the lower altitude favored the accumulation of acids associated with a less pleasant aroma. Finally, aldehydes and alcohols, which correlated positively between them, were negatively associated to acids. The reported results highlight that maturity stage is a crucial factor in determining the carob powder volatile profile, followed by altitude and genotype.

Zhang et al. conducted a comprehensive study about the crystal morphology, chemical composition and gene expression of cuticular wax (CW) of 12 different grape cultivars during berry development and storage. Morphological analysis of berries revealed high density of irregular lamellar crystal structures, which were correlated with the glaucous appearances of grape berry, while the compositional analysis indicated that the dominant wax compounds were triterpenoids, whereas alkanes were the most diverse class of compounds. The amounts of triterpenoids declined sharply after veraison, while the content of other compounds was almost constant during fruit development, indicating that CW were biosynthesized at the early stage. These findings suggest that CW contribute to the water preservation capacity of grape berries. The reduced water loss rate in cold-stored berries may result from the relatively high amounts of triterpenoids and low amounts of alkanes, respectively.

The expression patterns of wax-related genes were in consistent with the accumulation of wax compounds, underlining the crucial role of these genes in the wax formation in grape. These findings not only provide a better understanding of the characteristics of CW in grape, but also contribute to define the molecular basis of wax biosynthesis and regulation in grape. The study also demonstrated the role CW play in preserving water during grape berries storage.

The last study by Huang et al. investigated the efficacy of the combined application of malic acid and lycopene in delaying the development of browning in litchi fruit stored at 4 °C and 25 °C. Results revealed that the combined application of malic acid and lycopene was effective in preserving the membrane integrity and the contents of total phenols, anthocyanins and flavonoids, thus, hindering the browning development in litchi fruit, during the storage under both temperatures. The combined treatment also depressed the enzyme activity of polyphenol oxidase and peroxidase, both associated to oxidation of polyphenols. Additionally, correlation analysis showed that the level of phenols in the pericarp negatively influenced the browning index, and was positively associated to the DPPH radical scavenging capability.

The present Research Topic contributed to improve the knowledge on volatile organic compounds as markers of freshness, maturity stage, or genotypes, as well as to demonstrate the efficacy of new postharvest treatments to extend the shelf life of fresh vegetable products.

## Author Contributions

All authors listed have made a substantial, direct, and intellectual contribution to the work and approved it for publication.

## Conflict of Interest

The authors declare that the research was conducted in the absence of any commercial or financial relationships that could be construed as a potential conflict of interest.

## Publisher's Note

All claims expressed in this article are solely those of the authors and do not necessarily represent those of their affiliated organizations, or those of the publisher, the editors and the reviewers. Any product that may be evaluated in this article, or claim that may be made by its manufacturer, is not guaranteed or endorsed by the publisher.

